# Climate Trends and Consumption of Foods and Beverages by Processing Level in Mexican Cities

**DOI:** 10.3389/fnut.2021.647497

**Published:** 2021-07-21

**Authors:** Nancy López-Olmedo, Ana V. Diez-Roux, Carolina Pérez-Ferrer, Francisco-Javier Prado-Galbarro, Horacio Riojas-Rodríguez, Juan Rivera-Dommarco, Tonatiuh Barrientos-Gutierrez

**Affiliations:** ^1^Center for Population and Health Research, National Institute of Public Health, Cuernavaca, Mexico; ^2^Dornsife School of Public Health, Drexel University, Philadelphia, PA, United States; ^3^Center for Nutrition and Health Research, National Institute of Public Health, Cuernavaca, Mexico; ^4^National Council for Science and Technology, Mexico City, Mexico; ^5^General Director, National Institute of Public Health, Cuernavaca, Mexico

**Keywords:** climate trends, food consumption, NOVA, cities, Mexico

## Abstract

**Background:** Little is known about the potential impact of climate change on food systems and diet. We aimed to estimate the association of changes in rainfall and temperatures with consumption of unprocessed and processed foods among residents of Mexican cities by climate region.

**Methods:** We analyzed 3,312 participants of the 2012 Mexican National Health and Nutrition Survey with dietary intake and sociodemographic information linked to historical rainfall and temperature data collected by the Mexican National Weather Service. We classified foods as unprocessed, processed, or ultra-processed. We performed multilevel linear regression to estimate the association of annual change in rainfalls (for each 0.5 mm decrease) and temperatures (for each 0.1°C increase) at municipality level over the past 5 years with consumption of processed and unprocessed foods measured as the contribution to total energy intake. We investigated whether associations differed by climate region (tropical, temperate, and arid).

**Results:** Each 0.5 mm annual decrease in precipitation was associated with lower consumption of unprocessed foods and higher consumption of ultra-processed foods [mean differences in percent contribution to total energy intake −0.009% (95% CI: −0.019, < −0.001) and 0.011% (95% CI: 0.001, 0.021), respectively]. Each 0.1 degree Celsius annual increase in temperature was also associated with lower consumption of unprocessed and higher consumption of ultra-processed foods [mean differences in percent contribution to total energy intake was −0.03 (95% CI: −0.05, −0.01) and 0.03% (95% CI: <0.01, 0.05)]. When stratified by climate region these associations were only observed in tropical regions.

**Conclusions:** Decreases in rainfalls and increases in temperature were associated with lower consumption of unprocessed foods but higher consumption of ultra-processed foods, especially in tropical regions. Previous studies have established how food production affects the climate, our study suggests that climate change could, in turn, reinforce modern food production, closing a vicious circle with clear negative implications for planetary health.

## Introduction

Climate change refers to persistent changes in the mean or in the variability of key properties of climate including temperature and precipitation (1). Strong evidence indicates that food systems, which include the pre-production, production, post-production, consumption, waste, and disposal of food, contribute up to 30% of global gas emissions (2). In Mexico, agricultural activities are the third leading source of greenhouse emissions (3). It has also been established that if present trends in temperature and rainfalls continue, 6% of plants and 4% of vertebrates will be lost, and human life will be affected by water scarcity and reduced food availability (4). As a result, one of the United Nations Sustainable Development Goals is the improvement of sustainable food systems, especially sustainable agriculture (5). Moreover, in early 2019, the EAT-Lancet Commission developed targets for achieving healthy diets from sustainable food systems (6).

While researchers are increasingly studying the effects of food systems on climate change, less attention has been paid to the potential impact of climate change on food systems (2). There are different classifications of food systems, but two categorizations include traditional and modern food systems (7). Traditional food systems are typically characterized by short supply chains involving localized production, distribution, and consumption of unprocessed and minimally processed staple foods, with shorter shelf duration. In contrast, modern food systems are characterized by complex globalized networks, were many actors are involved in “long” supply chains, oriented toward maximizing efficiency, reducing costs, and increasing shelf durability of a wide variety of food types, largely ultra-processed foods. Traditional food systems are more vulnerable to local climate change, as they rely more heavily on production techniques that depend on predictable weather and seasonality, and lack the ability to store, modify or transport foods, factors in which modern food systems are highly effective (8). Thus, it is reasonable to expect that traditional food systems would be more affected by climate change than modern food systems. This implies that food systems and climate change could be involved in a vicious cycle, in which food production contributes to climate change, which in turn favors modern food systems that are worse for the environment (9), and intensify climate change. It would also imply, that as we move toward worse scenarios in climate change we could expect ultra-processed food consumption to increase, as food supply becomes more dependent on modern food systems, with important implications for both planetary and human health.

Urban food systems are of particular relevance for climate change, as more than 55% of the current human population live in cities (10). Urban food systems rely much more on modern food systems than rural areas, since local production of food in cities is limited; consequently, ultra-processed food consumption is much more frequent in cities than it is in rural areas (11). A large proportion of the Mexican population (48%) lives in cities with more than 100,000 inhabitants (12). Also, Mexico is a key food producer in the Latin American region and highly vulnerable to climate change (13). Food production in the country spans several climate zones that could be severely affected by small temperature or rainfall changes. A previous study suggests that the consequences of climate change could be different by climate region. It has been estimated that extreme climate events, including increases in temperature and rainfall could negatively impact food production in arid regions (14). Higher temperatures but reduced rain precipitations could also have negative consequences for food production in tropical regions. On the other hand, regions with temperate climate could benefit from increases in temperature and rainfall in the short- but not in the long-term (15).

We used rich dietary data available from a large representative survey conducted in Mexico to explore the association of changes in rainfall and temperature with food consumption, the final link in the food system chain. We hypothesized that recent reductions in rainfall and increases in temperature would be associated with more consumption of ultra-processed foods and beverages and less consumption of unprocessed foods and beverages, especially fruits and vegetables, across all climate regions. We also hypothesized that the magnitude of the changes in the consumption of unprocessed and processed foods would be higher in arid and tropical regions than in areas with temperate climates.

## Materials and Methods

### Study Design and Participants

The 2012 Mexican National Health and Nutrition Survey (ENSANUT for its Spanish acronym) is a cross-sectional, multistage, stratified, and cluster-sampled survey representative of urban and rural areas, at the national, regional, and state levels in Mexico (16). Briefly, trained interviewers collected information about sociodemographic, nutrition, and health characteristics from 96,031 people, between October 2011 and May 2012. Dietary information was obtained from a random subsample of 8% of the participants (*n* = 7,810) (17). We included non-pregnant and non-lactating individuals aged at least 1 year, living in cities as defined for the *Salud Urbana en América Latina* (SALURBAL) project. SALURBAL defines cities as urban agglomerations (clusters of municipalities) with more than 100,000 inhabitants (18). This resulted in 4,247 study participants with dietary information. Rainfalls and average temperatures measured in weather stations were obtained from databases collected by the Mexican National Weather Service (19). We linked the municipality of residence of participants in the 2012 ENSANUT to the weather stations in the same area. If there was more than one station in the municipality, we calculated the mean value of monthly rainfalls and temperatures. We excluded individuals living in municipalities without weather information in the area (*n* = 896) or without at least 2 years with complete monthly weather data during the 5-year period prior to the survey (*n* = 39). This left a total of 3,312 participants for analysis. These participants were nested in 164 municipalities with a median number of participants of 33 (interquartile range 17–67) per municipality.

### Study Measurements

#### Dietary Information

Interviewers collected dietary information using the 140-item Semi-Quantitative Food Frequency Questionnaire previously validated in Mexican population (20). Details on dietary intakes estimated with the Semi-Quantitative Food Frequency Questionnaire are available elsewhere (17). Briefly, for each food, participants reported the frequency as well as the number of standard portions they consumed over 7 days prior to the interview. We considered edible portions and density factors to obtain net grams of solid food consumed and milliliters consumed from beverages, respectively. Likewise, we calculated energy values using a Food Composition Table compiled by the Mexican National Institute of Public Health (21).

#### Study Outcome

We classified all foods and beverages according to NOVA, a food classification of four categories developed by Monteiro et al. (22), that is based on the physical, biological, and chemical techniques used after foods are separated from nature before they are consumed or prepared as dishes (22). For this study, we considered three food categories, the unprocessed and minimally processed food groups as a single category (hereinafter called “unprocessed”), the processed, and the ultra-processed food categories. We did not consider the category of culinary ingredients (e.g., salt, sugar, oils). Briefly, the category of unprocessed food refers to all plant- and animal-based foods which do not undergo any processing or those only altered by processes that include removal of unwanted parts, and drying, fractioning, filtering, roasting, boiling, non-alcoholic fermentation, pasteurization, refrigeration, chilling, freezing, placing in containers, and vacuum-packaging. The processed food category considers foods and beverages made by adding salt, oil, sugar, or other substances to those in the unprocessed group. The ultra-processed category includes foods and beverages made mostly or entirely from substances derived from foods and additives, with little if any intact unprocessed food (see Supplementary Table 1 for details about the foods and beverages included in each food category) (22). We calculated the contribution of each category of foods to the total energy intake (TEI).

#### Independent Variable

We used the monthly total rainfalls and monthly average temperatures for each municipality to calculate annual averages from the 5 years prior to the survey. Then we estimated the annual change in rainfalls and temperatures over the past 5 years in each municipality. To derive the annual change we used mixed-effects models including calendar time interacted with climate regions.

#### Potential Modifier

We georeferenced the municipalities by climate region using data from the National Biodiversity Information System (23). We considered the following main climate regions: tropical (including tropical and subtropical), arid (including semi-arid and arid) and temperate.

### Statistical Analysis

First, we examined the distribution of sociodemographic and climate characteristics in the overall sample and by climate region. Second, we performed multilevel multivariable linear regression models with individuals nested within municipalities to test the association between annual change in rainfalls (for each 0.5 mm decrease) and temperatures (for each 0.1°C increase) over the prior 5 years and consumption of processed and unprocessed foods (measured by their contributions to TEI) as measured in the ENSANUT 2012. Models were adjusted for climate regions (overall models) sex, age group, educational level (as a proxy of socioeconomic status), and rainfall or temperature change, as appropriate. We used the models below:

Overall model

$$\begin{array}{l} y_{ij}=\beta_{0}+\overset{n}{\underset{k=1}{\sum }}\beta_{k}W_{ki}+\overset{m}{\underset{l=1}{\sum }}\beta_{l}X_{lj}+\beta_{p}Z_{pj}+u_{j} \end{array}$$

Where *y*_*ij*_ is the contribution of unprocessed, processed or ultra-processed foods to the TEI for individual *i* in the municipality *j*, *W*_*i*_ is the set of covariables at the individual level (sex, age group, and educational level), *X*_*j*_ is the set of explanatory variables for the municipalities (temperatures and rainfalls), and *Z*_*j*_ are the climate region categories at the municipality level. The term *u*_*j*_ represents the residuals of level 2, for which it is assumed that they are independent and follow a normal distribution with mean 0 and variance σ^2^.

Interaction of temperatures and rainfalls with climate regions

$$\begin{array}{l} y_{ij}=\beta_{0}+\overset{n}{\underset{k=1}{\sum }}\beta_{k}W_{ki}+\overset{m}{\underset{l=1}{\sum }}{\beta_{l}X_{lj}}^{*}\beta_{p}Z_{pj}+u_{j} \end{array}$$

Where $X_{j}^{*}Z_{j}$ is the interaction of temperatures and rainfalls with climate regions

In additional analyses we explored consumption using different categorizations of foods. First we investigated consumption of unprocessed foods in more detail by subdividing it into three categories based on the environmental footprint of the foods: (1) Plant-based foods (fruits, vegetables, grains, and nuts); (2) seafood and poultry, (3) red meat and (4) milk (6). The first category of unprocessed foods, plant-based foods, was further subdivided into of (1) fruits and vegetables, and (2) grains and nuts (mostly grains), since the preservation of fruits and vegetables could be more susceptible to climate change than that of grains and nuts (24). Second, we investigated consumption of ultra-processed food in more detail by categorizing it as: (1) sugar-sweetened beverages, (2) high-energy dense foods (candies, cookies, pastries, sweet bread, ready-to-eat cereals, and salty snacks), (3) sweetened dairy drinks, and (4) convenient foods (processed meats, fast foods with processed meats, instant soups, and dressings). The category of high-energy was further analyzed by subdividing into four categories based on nutritional profile: (1) candies, (2) cookies, pastries, and sweet bread, (3) ready-to-eat cereals, and (4) salty snacks (see Supplementary Figure 1 for details about the food categories analyzed).

Statistical tests were two-tailed and considered significant under a 0.05 alpha. All analyses were carried out using Stata version 14 (StataCorp, Stata Statistical Software, Release 14, 2015).

### Ethics Approval and Consent to Participate

The 2012 ENSANUT was conducted according to the guidelines laid down in the Declaration of Helsinki and all procedures involving human subjects/patients were approved by the Ethics Committee of the Mexican National Institute of Public Health (project number 1033, approval 1108). Written informed consent was obtained from all subjects/patients under study. All the information used in the present study was obtained from de-identified secondary data.

## Results

Most of the participants lived in arid and tropical regions (43.1 and 38.7%, respectively). In the overall sample, 51.9% of the participants were women and 61.3% were adults (aged 20 years and over). Nearly half of the participants had primary education or less. The contribution of unprocessed, processed, and ultra-processed foods to TEI was 48.2, 6.7, and 37.3%, respectively. The distribution of food consumption by processing level as well as the distribution by sex and age group were similar across climate regions (Table 1).

**Table 1:** Characteristics of survey respondents included in analyses, overall and by climate region^a^.

	**Overall**	**Climate region**		
		**Tropical**	**Arid**	**Temperate**
Participants, *n* (%)	3,312	1,283 (38.7)	1,428 (43.1)	601 (18.1)
Sex				
Men	48.1	49.7	47.3	47.4
Women	51.9	50.3	52.7	52.6
Age group				
Preschool children (1–4 y)	6.9	7.3	6.9	6.6
School children (5–11 y)	13.8	14.6	13.9	13.1
Adolescents (12–19 y)	18	18.3	17.4	18.2
Adults (≥20 y)	61.3	59.7	61.8	62.1
Educational level				
Primary or less	41.7	43.5	39.7	42.2
Secondary	28.3	25.0	28.5	30.7
High school	13.7	15.8	13.6	12.3
Technical career or higher	16.3	15.7	18.2	14.8
Contribution of foods to total energy				
Intake				
Unprocessed	48.2	49.2	48.2	47.4
Processed	6.7	6.5	6.8	6.9
Ultra-processed	37.3	36.4	37.3	37.9
Municipalities, *n* (%)	164	65 (39.6)	53 (32.3)	46 (28.1)
Number of participants per municipality [median (p25, p75)]	33 (17, 67)	36 (17, 55)	53 (23, 84)	18 (10, 30)

a *Values are percentages, unless otherwise indicated*.

**Figure 1:**
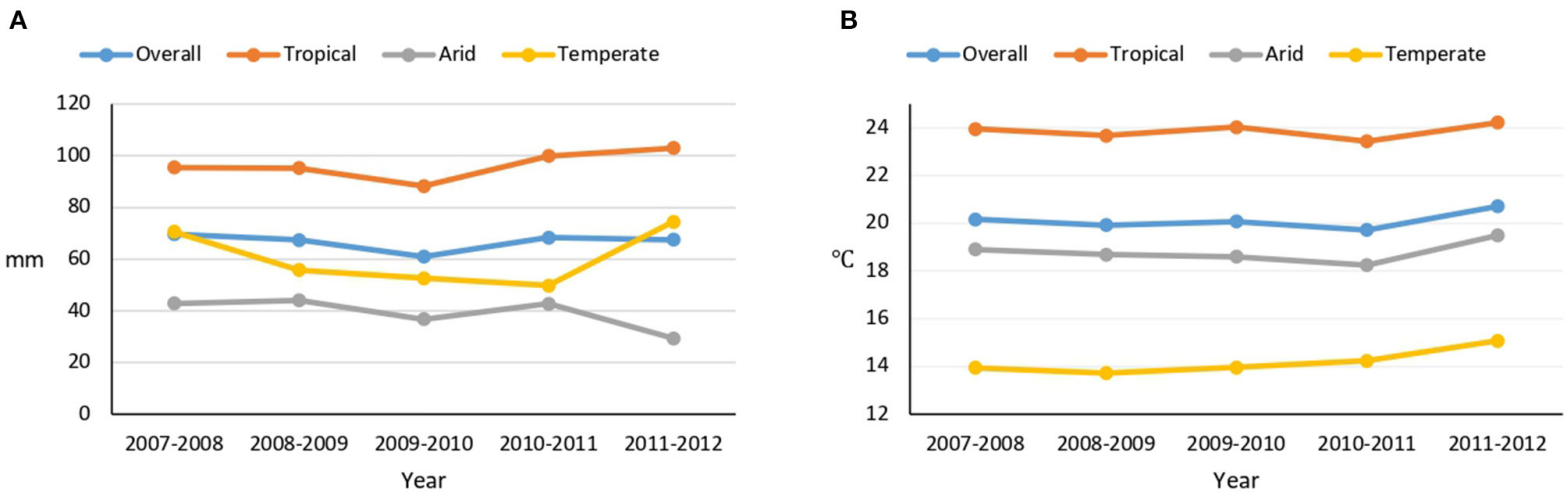
Mean annual rainfall **(A)** and temperature **(B)** in Mexican cities over the 5 years prior to the 2012 Mexican National Health and Nutrition Survey (2007–2008 to 2011–2012).

Overall, the mean annual rainfall decreased from 69.6 mm in 2007–2008 to 67.5 mm in 2011–2012 (an average 0.5 mm per year reduction). However, there were fluctuations in rainfalls during the period; overall rainfall decreased in arid regions and increased in tropical and temperate regions. The increase in rainfalls in temperate regions and the reductions in arid regions were particularly marked from 2010–2011 to 2011–2012 (Figure 1). Temperature increased in all regions, with an overall average increase of 0.1°C per year. The mean annual rainfalls and temperatures in each of the municipalities are presented in Supplementary Figure 2.

**Figure 2:**
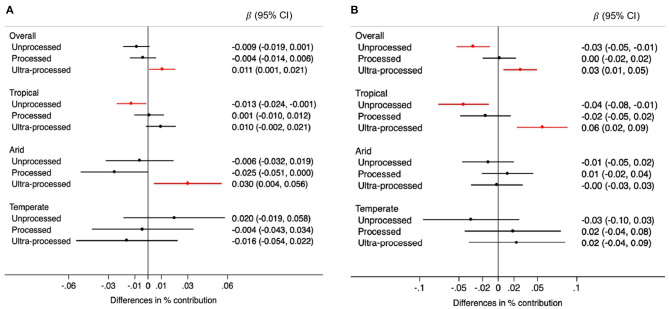
Differences in the percentage (95% CIs) contribution of foods by processing level to the total energy intake for each 0.5 mm annual decrease in rainfall **(A)** and for each 0.1°C annual increase in temperature **(B)** during the 5-year period prior to the survey, overall and by climate region. Values were obtained using multivariable multilevel linear regression models adjusted for climate region (overall models) sex, age group, educational level, and rainfall or temperature, as appropriate.

Overall, the contribution of unprocessed foods to TEI was 0.009% lower (95% CI: −0.019, 0.001) for each 0.5 mm annual decrease in rainfall over the prior 5 years. The opposite was observed for ultra-processed foods: their contribution was 0.011% higher (95% CI: 0.001, 0.021) for each 0.5 mm annual decrease in precipitations. When analyses were stratified by climate region this pattern (lower consumption of unprocessed foods and higher consumption of ultra-processed foods when rainfall decreased) was clearly observed in tropical areas. However, in arid regions a decrease in rainfall was associated with higher consumption of ultra-processed foods only. No statistically significant associations were observed in temperate areas but confidence intervals were very wide (Figure 2A). Changes in temperature were also associated with consumption patterns. Each 0.1 degree Celsius annual increase in temperature over the prior 5 years was associated with a 0.03% lower contribution of unprocessed foods to TEI (95% CI: −0.05, −0.01), and a 0.03% higher contribution of ultra-processed foods (95% CI: 0.01, 0.05). When analyses were stratified by climate region these associations were observed only in tropical regions (Figure 2B).

**Figure 3:**
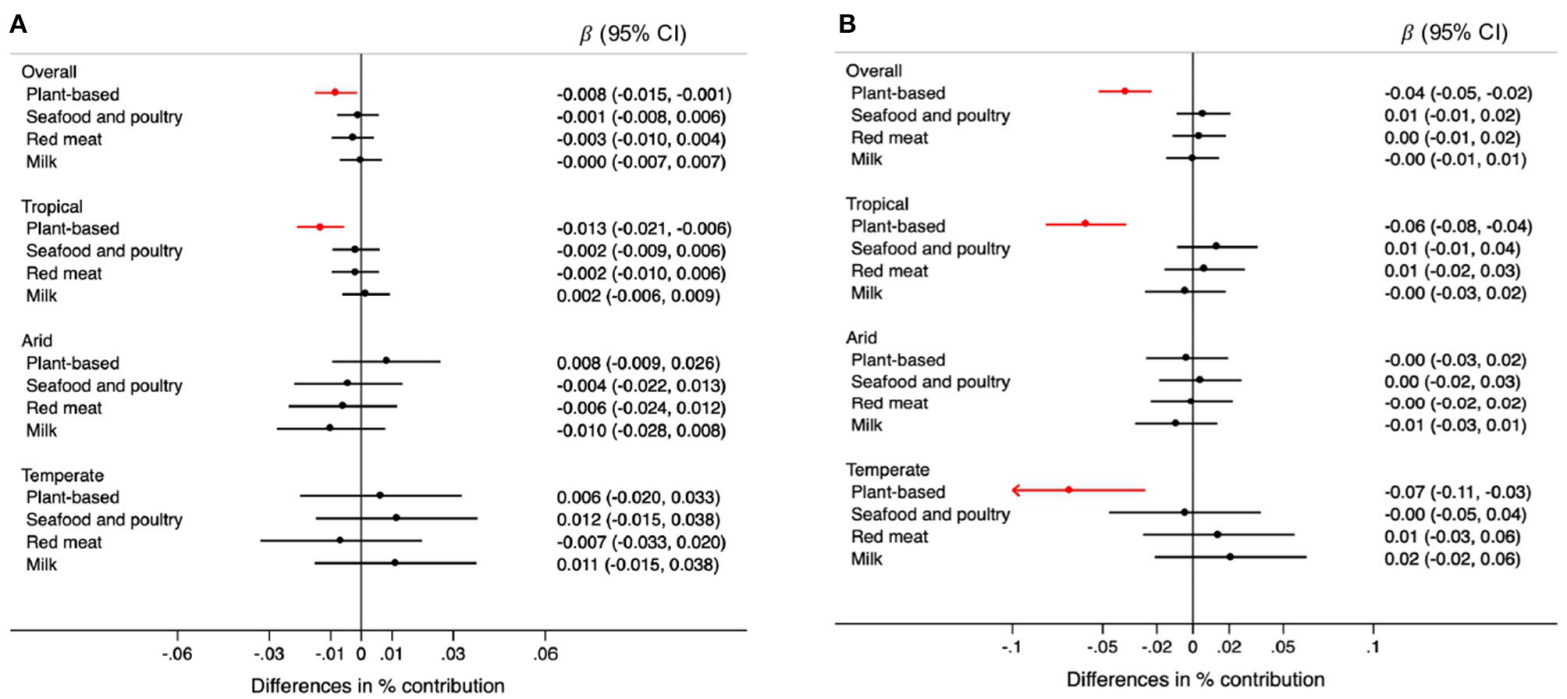
Differences in the percentage (95% CIs) of contribution of type of unprocessed foods and beverages to the total energy intake for each 0.5 mm annual decrease in rainfall **(A)** and for each 0.1°C annual increase in temperature **(B)** during the 5-year period prior to the survey, overall and by climate region. Values were obtained using multivariable multilevel linear regression models adjusted for climate region (overall models) sex, age group, educational level, and rainfall or temperature, as appropriate.

**Figure 4:**
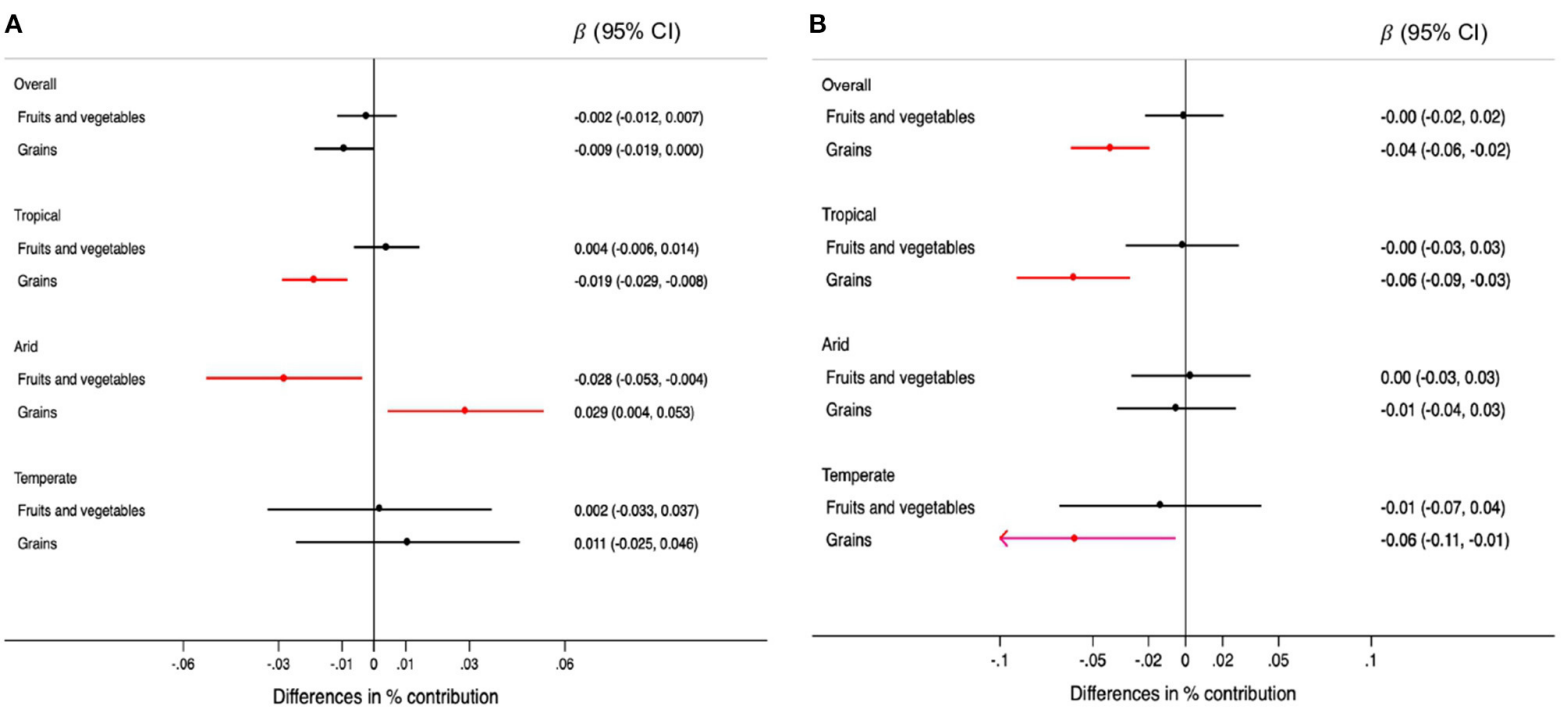
Differences in the percentage (95% CIs) of contribution of types of plant-based foods to the total energy intake for each 0.5 mm annual decrease in rainfall **(A)** and for each 0.1°C annual increase in temperature **(B)** during the 5-year period prior to the survey, overall and by climate region. Values were obtained using multivariable multilevel linear regression models adjusted for climate region (overall models) sex, age group, educational level, and rainfall or temperature, as appropriate.

For each 0.5 mm decrease in rainfall, the contribution of plant-based foods was 0.008% lower (95% CI: −0.015, −0.001) in the overall sample. This association was observed only in tropical regions (Figure 3A). Specifically, grains were the plant-based food for which consumption was lower when rainfall decreased in tropical regions (Figure 4A). Each 0.1 degree Celsius annual increase in temperature was associated with a 0.04% lower contribution of plant-based foods to TEI (95% CI: −0.05, −0.02). This association was observed in regions with tropical and temperate climate (Figure 3B). Likewise, grains were the plant-based food inversely associated with temperatures, overall and in tropical and temperate regions (Figure 4B).

**Figure 5:**
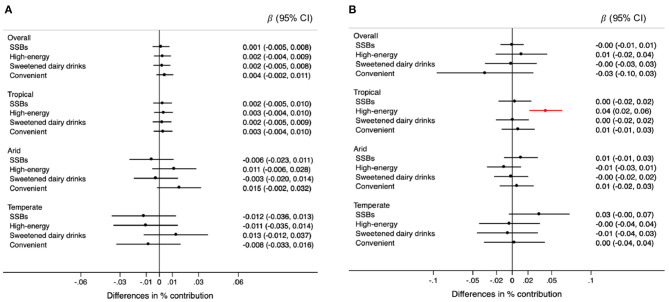
Differences in the percentage (95% CIs) of contribution of types of ultra-processed foods and beverages to the total energy intake for each 0.5 mm annual decrease in rainfall **(A)** and for each 0.1°C annual increase in temperature **(B)** during the 5-year period prior to the survey, overall and by climate region. Values were obtained using multivariable multilevel linear regression models adjusted for climate region (overall models) sex, age group, educational level, and rainfall or temperature, as appropriate. High-energy category includes candies, cookies, pastries, sweet bread, ready-to-eat cereals, and salty snacks. Convenient category includes processed meats, fast foods with processed meats, instant soups, dressings.

**Figure 6:**
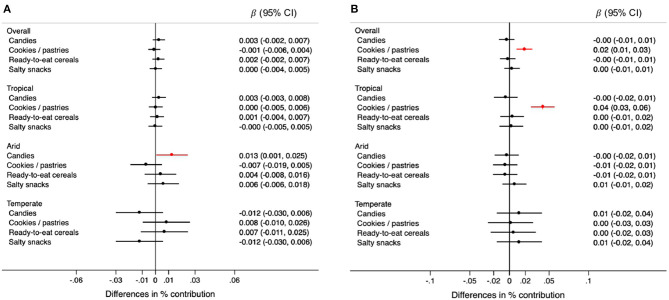
Differences in the percentage (95% CIs) of contribution of types of high-energy ultra-processed foods to the total energy intake for each 0.5 mm annual decrease in rainfall **(A)** and for each 0.1°C annual increase in temperature **(B)** during the 5-year period prior to the survey, overall and by climate region. Values were obtained using multivariable multilevel linear regression models adjusted for climate region (overall models) sex, age group, educational level, and rainfall or temperature, as appropriate.

Changes in rainfall were not associated with consumption of any specific type of ultra-processed food (Figure 5A). However, changes in rainfall were associated with higher consumption of candies in arid regions (Figure 6A). For each 0.1 degrees Celsius annual increase in temperature, the contribution of high-energy foods was 0.04% higher (95% CI: 0.02, 0.06) only in tropical climates (Figure 5B). Specifically, cookies, pastries, and sweet bread were positively associated with temperature, in the overall sample and tropical regions (Figure 6B). Although not quite statistically significant, the increase in temperature was associated with higher consumption of sugar-sweetened beverages in arid and temperate regions (difference in contribution to TEI <0.01%, 95% CI: −0.02, 0.02 and 0.01%, 95% CI: < -0.01, 0.07, respectively) (Figure 6B).

## Discussion

We investigated whether recent (5 year) changes in rainfall and temperature were associated with the consumption of unprocessed, processed, and ultra-processed foods and beverages among residents of Mexican cities in different climate regions. We found that decreases in rainfall were associated with lower consumption of unprocessed foods and higher consumption of ultra-processed foods. We also found that increases in temperature were associated with lower consumption of unprocessed foods and higher consumption of ultra-processed foods. The associations of rainfall and temperature changes with consumption were statistically significant in tropical regions.

The findings of lower consumption of unprocessed foods associated with decreases in rainfall may be driven at least in part by lower production of grains at lower precipitations. Rainfall is one of the most important factors for the growth of cereals, as already observed in studies in Mexico and other countries (25–29). Specifically, rainfed corn is a fundamental component of agriculture in Mexico and a staple food among Mexicans (11, 28, 30); corn tortilla, a minimally processed food, represented 20% of TEI among adults in 2012 (11). Lower production of grains, such as corn, can, in turn, lead to lower availability and higher food prices and, therefore, lower consumption of these types of unprocessed or minimally processed foods (31, 32). Given the downward trend in rainfalls in arid climates from 2007 to 2012, increases in precipitations could benefit food production, especially grains, in these regions. It would also be expected that the production and consumption of fruits and vegetables were affected by the weather. However, most fruits and vegetables produced in Mexico are high-value export crops produced in irrigated lands (33).

A reduction in the contribution of unprocessed foods to TEI at lower precipitations can lead to a higher contribution of ultra-processed foods by substitution. The associations of less rainfall with lower consumption of unprocessed foods and higher consumption of ultra-processed foods were observed primarily in tropical regions. This can be explained by the key role of subtropical regions, which have the most productive areas of rainfed cornfields in the country (34). Moreover, in our data the regions with the highest increase in rainfall from 2007 to 2012 were the tropical regions. Therefore, it is likely that favorable weather conditions led to higher production of grains in tropical regions and, therefore, higher consumption of this type of food.

Although we found adverse effects of decreases in precipitation on food consumption patterns, we recognize that heavy precipitations could have negative effects on food production. At the same time water deficits and droughts are the largest causes of crop losses (35). In the United States, drought was related to 41% of crop losses, while excess water was considered responsible for 16% of the yield loss (36).

Higher temperatures can also lead to lower production of grains. Reduced food production at higher temperatures, especially of cereals, has been observed in European and African countries (25, 37, 38). This may be because temperature largely determines the duration of plant growth (39). Specifically, higher temperatures may accelerate crop development and reduce grain filling duration (40), and the shorter duration of the grain filling period has been identified as the main driver of grain yield decreases. Lower production of grains could lead to increase in the price of these types of food and, in turn, to consumers altering their food choices (41, 42). In the specific case of corn, model estimates suggest that for every 1°C increase in temperature, there is about 10% yield reduction (35). High temperatures can also have negative effects on pollen survivability (35). Another explanation for the link between higher temperatures and lower consumption of unprocessed foods may be that unprocessed foods spoil faster during transport and at stores with high temperatures leading to less availability.

The higher consumption of ultra-processed foods that we observed when temperature increases may result simply from the lower availability and higher price of unprocessed foods as a consequence of their lower production. Another potential explanation of the higher consumption of ultra-processed foods at higher temperatures is that elevated temperatures may also increase foodborne pathogens in the pathway between farm and consumer. Therefore, individuals may prefer foods and beverages that are packaged, such as most of the ultra-processed foods. Moreover, the preference for certain foods, such as liquid foods, can increase with higher temperatures. Although the results were not statistically significant, we observed higher consumption of sugar-sweetened beverages at higher temperatures in arid and temperate regions. Though evidence is limited, some studies have reported higher intake of sugar-sweetened beverages in the summer than in the winter (43, 44), which suggests that the consumption of this type of beverages is higher as the temperature increases. We also observed that the consumption of high-energy ultra-processed foods, specifically cookies, pastries, and sweet bread, was higher at higher temperatures in tropical regions. Given that we also found lower consumption of grains at higher temperatures, a possibility is that the consumption of grains is substituted by ultra-processed foods that contain grains or derived products from grains. It could be argued that the production of processed foods would also be affected by higher temperatures and decreases in rainfalls since the production of these types of food products implies the use of substances derived from natural food. However, processed foods, and especially ultra-processed foods, are massively produced by using raw foods that come, for example, from intensive irrigated monocultures of only a few plant varieties (45). Therefore, processed foods could be less affected by climate change.

This study has limitations that we acknowledge. First, we used cross-sectional information on dietary patterns, limiting our ability to establish a clear causal link. Longitudinal diet data would be needed to directly link changes in temperature and precipitation to changes in diet. Reverse causality could pay a role if consumption patterns reflected by 2012 ENSANUT are a proxy of historical industrial development, which is known to produce climate change. However, if reverse causality were the main explanation for our findings, we would not expect to see such large heterogeneity across climate regions: increases in ultra-processed foods consumption should be linked to precipitation and temperature across all climates. Instead, our findings suggest that certain climates are more prone to induce changes in food consumption as a result of changes in precipitation and temperature, which suggests a climate-related mechanism rather than an industrial change mechanism. Second, the changes in the contribution of foods by processing level estimated are small, which suggests that local food consumption is limited. However, decreases in rainfall of 10 mm, as observed in arid regions from 2007–2008 to 2011–2012, would translate into reductions in the intake of unprocessed foods of ~40 kcal per day, while the intake of ultra-processed food would increase ~40 kcal per day (based on a 2,000 kcal diet). This impact is not negligible given that every change of energy intake of 25 kcal will result in an eventual bodyweight change of about 1 kg in adults with overweight (46). Third, we cannot rule out that some statistically significant results can be due to chance given the multiple testing. However, we note that our findings are consistent particularly for tropical regions. Finally, although food frequency questionnaires have been shown to provide valid and reliable dietary data (47), they remain imperfect and may have resulted in misclassification of foods by processing level. Despite these limitations, our analyses provide valuable initial evidence suggesting a link between climate change and diet by using a large and diverse sample with detailed dietary data.

## Conclusion

We observed lower consumption of unprocessed foods and higher consumption of ultra-processed foods after decreases in rainfall and increases in temperature. Climate change affects a wide range of health outcomes and, our findings, although exploratory, highlight the potential impact of climate change in diet. Most importantly they highlight the possibility of a vicious cycle by which climate modifies diet and leads to changes in food demand that in turn promote climate change. These findings further highlight why global action on climate change to protect our environment and our health is urgent. Also, policies are needed to increase consumer awareness about the potential negative effects of ultra-processed foods on health and the environment and how climate change could affect healthy food choices through their availability and accessibility.

## Data Availability Statement

Publicly available datasets were analyzed in this study. This data can be found at: https://ensanut.insp.mx/encuestas/ensanut2012/descargas.php; https://smn.conagua.gob.mx.

## Ethics Statement

The studies involving human participants were reviewed and approved by Ethics Committee of the Mexican National Institute of Public Health. Written informed consent to participate in this study was provided by the participants' legal guardian/next of kin.

## Author Contributions

NL-O and TB-G: study design. NL-O: data curation, drafting, and editing of the manuscript. AD-R, CP-F, and F-JP-G: contribution of the study design. HR-R: contribution to the analysis of temperature and rainfall. All authors contributed to the critical revision of the manuscript and approved the submitted version.

## Conflict of Interest

The authors declare that the research was conducted in the absence of any commercial or financial relationships that could be construed as a potential conflict of interest.

## Abbreviations

ENSANUT: Mexican National Health and Nutrition Survey (for its Spanish acronym)
SALURBAL: Salud Urbana en América Latina (Urban Health in Latin America) project
TEI: total energy intake.
