# Supplementary material for: Climate Trends and Consumption of Foods and Beverages by Processing Level in Mexican Cities
Source: Front Nutr. Author manuscript; Available in PMC 2021 Aug 24. (PMC8334732; doi:10.3389/fnut.2021.647497)
Supplement: Supplementary file 1 [file EMS131092-supplement-Supplementary_file_1.docx]

**Supplementary Table 1.** Examples of foods and beverages included in each category

| **Food category** | **Examples** |
| --- | --- |
| Unprocessed | Coffee, tea or 100% fruit juice without sugar, natural yoghurt, *aguas frescas* without sugar, natural water, soups, raw fruit, raw vegetables, raw legumes, tortillas, frozen vegetables |
| Processed | Mature cheese, nuts, canned vegetables, crystallized fruit, canned fish |
| Ultra-processed | Sodas, industrialized beverages, flavored yoghurt, chocolate, candies, salty snacks, cakes, jelly, ice cream, sorbet, microwave popcorn, industrialized pastries and donuts, yoghurt *light,* cookies, instant soups, yoghurt drink, margarine, vegetable shortening, mayonnaise, ketchup, instant sauces, flavored milk, sausages |

**Supplementary Figure 1.** Food categories

**A**

**B**

**C**

**Supplementary Figure 2.** Mean annual rainfall and temperature in Mexican in each urban municipality in tropical (A), arid (B) and temperate (C) regions over the 5 years prior to the 2012 Mexican National Health and Nutrition Survey (2007-2008 to 2011-2012). Each line represents the mean annual rainfalls and temperatures in each municipality (n=164).
